# Reeling from news that reelin defends the brain against Alzheimer’s

**DOI:** 10.1016/j.xcrm.2023.101111

**Published:** 2023-07-18

**Authors:** Edmond N. Mouofo, Tara L. Spires-Jones

**Affiliations:** 1 Centre for Discovery Brain Sciences and UK Dementia Research Institute at the University of Edinburgh, Edinburgh, UK

## Abstract

In a recent study, Lopera and colleagues investigate a person with extreme resilience to autosomal-dominant familial Alzheimer’s disease, which they attribute to a rare variant in the *RELN* gene encoding reelin.^1^

## Main text

Alzheimer’s disease (AD) is the most common cause of dementia, constituting a rapidly growing financial and personal burden as populations of the world age. The pathological hallmarks of AD include accumulation of amyloid-β in amyloid plaques, tau aggregation in neurofibrillary tangles, and brain atrophy resulting from neuron and synapse loss. Understanding factors that confer brain resilience to AD is at the forefront of research. Around 30% of older adults exhibit neuropathological features of AD without cognitive impairment,^2^ indicating that even in the face of pathology, the brain is capable of resisting cognitive decline.

Based on the age of onset and genetic predisposition, AD is classified into sporadic (late-onset) AD or familial (early-onset) AD. Even though sporadic AD accounts for 95% of all cases, our understanding of AD has been greatly influenced by the study of families with AD-causing autosomal-dominant mutations in presenilin 1 (*PSEN1*), presenilin 2 (*PSEN2*), and amyloid precursor protein (*APP*). Lopera and team have characterized more than 1,200 people with the *PSEN1* E280A mutation from the world’s largest autosomal-dominant AD kindred and used this powerful cohort to look for genetic factors that can delay the age of onset of dementia in the hopes of finding pathways that confer resilience.^1^ The protective *APOE3* “Christchurch” variant was discovered by this group in 2019. In a woman in the Columbian kindred, this variant was associated with an approximate 30-year delay in developing AD.^3^ In the May 2023 issue of *Nature Medicine*, the same group report another gene variant associated with AD resilience. The team described a Colombian brother and sister carrying a rare variant in the *RELN* gene (H3347R coined as *RELN-COLBOS* after the Columbia-Boston biomarker research study) who showed delayed onset of autosomal-dominant Alzheimer disease despite carrying the *PSEN1* E280A mutation known to cause mild cognitive impairment (MCI) by around age 44 and AD type dementia by age 49.^1^ The male patient with the *RELN-COLBOS* variant was functionally independent and cognitively intact until age 67 when he was diagnosed with MCI. This corresponds to an approximate 20-year delay in symptom onset compared to the rest of the cohort. He progressed to mild dementia by age 72 and died at age 74.

One potential mechanism underlying the observed resistance to AD in the *RELN-COLBOS* carrier is via delaying accumulation of tau pathology. Previous work showed that reelin signaling via Dab1 induces GSK3β activation and tau phosphorylation.^4^ *In vitro* and *in vivo* studies in the Lopera paper showed that *RELN-COLBOS* increased levels of phosphorylated Dab1. A PET study of the male *RELN-COLBOS* carrier revealed less tau pathology in the entorhinal cortex, cingulate cortex, and precuneus than age-matched *PSEN1*-E280A carriers with MCI and dementia despite higher levels of cortical amyloid beta plaque burden compared to younger impaired PSEN1-E280A carriers in the study. The idea that *RELN-COLBOS* influences tau pathology is supported by the mouse data in Lopera et al. showing slightly less intense pathological tau staining in human tau transgenic mice with the mouse equivalent of the *RELN-COLBOS* mutation. However, neuropathological examination confirmed the *RELN-COLBOS* carrier had severe AD pathology (Braak stage VI), so although the *RELN-COLBOS* variant substantially delayed onset, it did not prevent typical neuropathological progression by end stage. Added to that, there were only three mice per group in the tau pathology study so the effects *RELN-COLBOS* on tau accumulation require further substantiation.

This report also highlights potential sex differences in resilience with this variant. The woman with the same *PSEN1* E280A mutation and *RELN-COLBOS* variant was less protected than her brother (delayed onset by 12 years). However, her earlier dementia onset could be attributed to various comorbidities including brain trauma, hypertension, and depression. Potential sexually dimorphic effects of *RELN-COLBOS* variant were further investigated in the new mouse model in this study. In the *RELN-COLBOS* mice, the males had a small but statistically significant increase in phosphorylated Dab1 levels compared to the females (however, this was normalized to a housekeeping protein instead of to total Dab1 raising the possibility that Dab1 levels themselves were changed, not the phosphorylation). Also, male human tau transgenic mice expressing the *RELN-COLBOS* allele had rescued abnormal limb-clasping phenotype. However, like the human study, this mouse study had caveats—small sample sizes and behavior only in male animals—so more work will be needed to confirm whether the *RELN-COLBOS* variant has meaningful sexually dimorphic effects on resilience to AD.

One interesting potential mechanism linking reelin variants to AD resilience that was not explored in the Lopera paper is potential synaptic protection. The activation of Dab1 by reelin is known to influence synaptic function by modulating post-synaptic receptor activity.^4^ Loss of synapses is the strongest pathological correlate of cognitive decline in AD, and preservation of robust synaptic structure and function has been associated with resilience to AD.^5^^,^^6^ We have previously observed strong links between genetic risk factors *APOE4* and clusterin and synaptic pathology accumulation^7^^,^^8^; thus, it will be very interesting in future studies to look at potential synaptic protection in *RELN-COLBOS* carriers who have amyloid pathology in their brains.

Genetic contributors to sporadic AD (so-called “risk-genes”) have also informed understanding about mechanisms of brain resilience and vulnerability to AD with variants like *APOE2* (apolipoprotein E epsilon 2) reducing risk while *APOE4* is associated with a large increase in risk of developing AD. Recent genetic studies in sporadic AD have reported multiple gene variants and pathways associated with resilience.^9^ Further support of the importance of the *RELN-DAB1* pathway in AD was published last year by Bracher-Smith and colleagues who found a novel genome-wide significant locus associated with AD mapping to *DAB1* in *APOE4* homozygots.^10^

This study by Lopera and colleagues shows remarkable resilience to familial Alzheimer’s disease in two people with the newly described *RELN-COLBOS* variant. Together, this study and the Bracher-Smith paper provide strong genetic support for an important role of reelin-Dab1 signaling in resilience to AD pathology. Further exploration of mechanisms using the important mouse model developed by Lopera and team will hopefully lead to new avenues to prevent, delay, or treat Alzheimer’s disease by enhancing the brain’s brilliant powers of resilience.
